# Effect of copper and silver ions on sequence and DNA methylation changes in triticale regenerants gained via somatic embryogenesis

**DOI:** 10.1007/s13353-022-00717-9

**Published:** 2022-08-19

**Authors:** Katarzyna Anna Pachota, Renata Orłowska

**Affiliations:** Plant Breeding and Acclimatization Institute – National Research Institute, Radzików 05-870 Błonie, Poland

**Keywords:** CG, CHG, CHH, Copper, Epigenetic variation, Genetic variation, Immature zygotic embryos, metAFLP, Silver

## Abstract

Somatic embryogenesis is a plant regeneration method that can be exploited in tissue culture systems for a variety of tasks, such as genetic modification or the selection of somaclones with advantageous characteristics. Therefore, it is crucial to create efficient regeneration procedures and comprehend how medium components affect regeneration effectiveness or the degree of variation created in plant tissue cultures. The level of tissue culture-induced variation in triticale regenerants was examined in the current study in relation to the concentration of copper and silver ions in the induction media as well as the length of time immature zygotic embryo explants were incubated on these media. The high degree of variation (45%) revealed by the methylation-sensitive amplified fragment length polymorphism approach for estimating variation included 38% DNA sequence alterations, 6% DNA demethylation, and 1% de novo DNA methylation. Different levels of variance were found in relation to various DNA sequence settings. The CHG context had the most alterations, whereas CG experienced the fewest; sequence variation predominated in each sequence context. Lower copper ion concentrations showed the most variance. However, it could not be connected to the duration of in vitro culture or the effect of silver ions. Accordingly, we think that altering the concentration of copper ions in the induction medium may throw off the equilibrium of the metabolic processes in which copper is involved, resulting in tissue culture-induced variation.

## Introduction

Plant tissue culture (PTC) methods take advantage of the induced plant cell totipotency, which finds its outstanding reflection in the phenomenon of somatic embryogenesis (SE). Somatic embryogenesis is the multi-step developmental process in which somatic cells of plant explants differentiate into bipolar structures known as somatic embryos. Induction of SE can occur by acting on the explants with stress (Zavattieri et al. 2010) or through appropriate growth regulators (Méndez-Hernández et al. 2019). In addition, the success of plant regeneration through SE is also influenced by genotype (Fiuk and Rybczyński 2008), type and age of explant (Atak et al. 2008), condition of donor plants (Dahleen 1999), the composition of culture media (Dahleen and Bregitzer 2002), and especially type and concentration of growth regulators (Przetakiewicz et al. 2003) or carbon source (Ganesan and Jayabalan 2005) as well as the release of organic molecules by the explants into the culture medium (Nic-Can et al. 2015). Furthermore, depending on the mode, somatic embryo formation can occur either directly (without the callus phase) (Eudes et al. 2003) or indirectly (Vega et al. 2009), when somatic embryos are formed on the produced calli.

Over 60 years of studies on the phenomenon of SE has led to developing protocols for plant regeneration for many species, including cereals. In vitro, culture methods allowing obtaining plants via the SE pathway have also been elaborated for triticale, a new, and synthetic crop with increasing importance for agriculture and plant breeding. Successful induction of SE in triticale was first reported for immature inflorescence-derived callus cultures (Eapen and Rao 1985) and then in callus cultures obtained from immature (Zimny and Rybczyński 1985) and mature zygotic embryos (Padmaja et al. 1992). When it comes to the last two types of explants, embryogenic callus from immature embryos is more likely to produce somatic embryos than tissue grown from mature embryos (Vikrant and Rashid 2001). Although, in the case of triticale, more emphasis seems to have been put on systems of androgenic embryogenesis to obtain plants that are doubled haploids (DH) (Oleszczuk et al. 2004), the induction of SE with the use of zygotic embryos as explant is constantly practiced (Machczyńska et al. 2015).

Regenerants obtained by SE should be morphologically and genetically identical to the donor plant. However, this assumption is not always valid. Some changes (phenotypic, cytological, genetic) are observed in regenerants (de la Puente et al. 2008). The changes are defined by the common term somaclonal variation (Larkin and Scowcroft 1981) or tissue culture-induced variation (TCIV) (Machczyńska et al. 2014). The first term describes changes affecting regenerants (Eeckhaut et al. 2020) and sometimes also their generative progeny (Zehr et al. 1987), while the second one refers only to changes affecting regenerants (Orłowska et al. 2021b). The causes of variation induced by in vitro culture are complex (Bednarek and Orłowska 2019; Bednarek et al. 2021); indeed, the components forming the culture media (Elena and Ginzo 1988; Immonen 1996), time (Etienne and Bertrand 2003), and conditions of PTC can be the source of variation. For example, among the culture media components, micronutrients such as copper or silver seem to influence the course of SE and, as recently shown, genetic and epigenetic changes (Orłowska et al. 2021b).

At a concentration of 0.1 M, copper sulfate (CuSO_4_) is one of the main components of the MS medium, and it exhibits positive effects on callus production and regeneration (Malik et al. 2021). Copper ions have been widely used in PTC to promote plant regeneration via SE (Dordević et al. 2019) and androgenesis pathways (Warchoł et al. 2021). In the latter case, copper ions reduced the production of albino plants (Grauda et al. 2014). Also, CuSO_4_ in a concentration of 0.1–100 μM significantly improved the regeneration of triticale shoots (Purnhauser and Gyulai 1993). On the other hand, the concentration of CuSO_4_ from 1 to 80 μM had a positive effect on the induction of SE and plant regeneration and, in the case of androgenesis, it decreased the number of albino regenerants (Cho et al. 1998; Jacquard et al. 2009). However, the concentration of 100 μM had a toxic effect and suppressed the formation of embryogenic calli, adversely influenced regeneration, and promoted the formation of albino wheat plants (Miroshnichenko et al. 2021).

Silver nitrate (AgNO_3_) (Rojas-Lorz et al. 2019), silver thiosulfate (Ag_2_S_2_O_3_) (Diab 2017), and silver nanoparticles (AgNPs) (Malik et al. 2021) are all added to in vitro culture media. Their primary function is to accelerate the callogenesis process and improve plant regeneration. Silver nitrate, usually added to the culture media at a concentration of 6–88 μM, impacts SE, shoot formation, efficient root growth, and organogenesis, prerequisites for successful genetic transformation (Paladi et al. 2017). The use of AgNO_3_ improved SE in wheat species *Triticum durum* (a more than 22-fold increase) (Fernandez et al. 1999) and *Triticum aestivum* (Wu et al. 2006), barley (Orłowska et al. 2020), maize (Carvalho et al. 1997), and rice (Ghobeishavi et al. 2015). Silver nitrate has been shown to mediate the inhibition of ethylene (Beyer 1976), a plant hormone involved in many developmental processes, including fruit ripening, abscission, senescence, growth, and flowering (Hobson et al. 1984). As an ethylene inhibitor, AgNO_3_ has significantly improved shoot regeneration in wheat (Purnhauser et al. 1987) and counteracted the aging process of callus of this species (Wu et al. 2006).

Time is another factor that may impact somatic embryogenesis. The extended duration of in vitro culture increases the number of mutations in regenerated plants (Duncan 1996), which may involve an increased mutation rate (Murashige and Nakano 1965) and sequential accumulation of mutations over time (Kaeppler et al. 2000). Thus, prolonged tissue culture time resulted in increased variation, manifested in different phenotypic variants (Armstrong and Phillips 1988). Also, increased callus induction time during SE decreases plant morphogenic potential and increases the numbers of albino plants and calli (Wen et al. 2004). It was documented that DNA methylation changes in regenerants may be related to time on the tissue culture run (Bednarek and Orlowska 2020).

Assessment of TCIV variation can be performed at different levels using a range of techniques, from the use of morphological characteristics of the clones (Eeckhaut et al. 2020) through the use of biochemical methods (Thomas et al. 2006) to molecular methods (Bednarek et al. 2007; Orłowska and Bednarek 2020; Patzak et al. 2021). The development of molecular techniques such as methylation-sensitive amplified fragment length polymorphism (metAFLP) has made it possible to characterize the variation induced by in vitro cultures at the level of DNA sequence and its methylation in one analysis. The metAFLP exploits the properties of restriction enzymes which are sensitive to the presence of methylated cytosine in or around the cutting site. The metAFLP method utilizes the Acc65I and KpnI isoschizomers to recognize the 5′-GGTACC-3′ sequence. Acc65I does not hydrolyze a DNA strand if the cytosine is methylated. On the other hand, the restriction enzyme KpnI cuts the DNA strand regardless of the presence of cytosine methylation. In the metAFLP technique, the differences between DNA profiles result from cutting the DNA of the donor plant and the regenerant with two pairs of restriction enzymes, namely Acc65I/MseI and KpnI/MseI. The method allows the estimation of changes both qualitatively and quantitatively. Using the isoschizomers permits quantitative assessment of TCIV and its components (sequence variation-SV, demethylation-DMV, and de novo DNA methylation-DNMV). In addition, the development of the method enabled the estimation of changes in specific DNA nucleotide sequences (hereafter referred to as sequence contexts) reflecting symmetric (CG and CHG) and asymmetric (CHH) cytosine methylation (H stands for A, C, T).

Although the function of copper and silver ions is relatively well studied concerning obtaining regenerants in PTC, including cereal ones, there is a lack of information on how these ions, in combination with various incubation times of immature zygotic embryos on induction medium (IM), can influence TCIV in triticale regenerants obtained by SE. Thus, the aim of this study was to evaluate the impact of copper and silver ions and incubation time of explants on TCIV in triticale regenerants obtained via SE in immature zygotic embryo cultures.

## Material and methods

### Acquisition of donor plants

The cultivar T28/2 of winter triticale (X *Triticosecale* spp. Wittmack ex A. Camus 1927) derived from cv. Presto × cv. Mungis cross provided by dr Sylwia Oleszczuk (Plant Breeding and Acclimatization Institute-NRI, Radzików, Poland) served to prepare donor plants via in vitro anther culture. The precise procedure for getting donor plants was previously published (Pachota et al. 2022). Simply put, we first obtained regenerants that were DH in anther culture. The final donor plants in the experiment were then obtained as the generative progeny of DH regenerants. Twenty donor plants were used to conduct additional research on triticale plant regeneration via SE from immature zygotic embryos under various in vitro growth conditions.

### Plant regeneration via somatic embryogenesis

Twenty donor seedlings were cultivated in a growth chamber at 16/12 °C (day/night). Photoperiod (16 h/8 h; light/dark) and light intensity (190 μE m^−2^ s^−1^) were maintained using high-pressure sodium lamps. After vernalization at 4 °C for 6 weeks under short-day photoperiod (8 h/16 h) in 20 μE m^−2^ s^−1^ light intensity plants were grown in greenhouse conditions. Spikes with unripe caryopses were harvested after 12–16 days of self-pollination. The caryopses removed from the tillers were surface-sterilized with 70% ethanol for 1 min and then transferred to 10% sodium hypochlorite solution (NaOCl) for 20 min. The caryopses were then thoroughly rinsed four times with distilled water. Immature zygotic embryos were dissected from the disinfected caryopses and plated on an MS induction medium (Murashige and Skoog 1962) with 2 mg l^−1^ 2,4-dichlorophenoxyacetic acid. Nine variants of tissue culture (trials: T1–T9) included IM with various concentrations of CuSO_4_ × 5H_2_O (0.1, 5, 10 μM) and AgNO_3_ (0, 10, 60 μM), and the incubation time (35, 42, 49 days) of immature embryos on these media (Table 1). The combination of the factors tested (Cu, Ag, time), as well as the concentration levels and the number of days, was determined according to the design of experiment principles based on Taguchi’s orthogonal arrays (Taguchi 1986). The induction step’s length (incubation time) covered the time from plating embryos on IM to calli collection and transferring them on regeneration media. Trial T1 was considered a control. Immature zygotic embryos were incubated on IM at 26 °C under a photoperiod of 16 h/8 h (light/dark). After several days, first calli and subsequent somatic embryos were recorded and transferred on a solid regeneration medium 190–2. Calli and embryos were incubated at 26 °C under 16 h/8 h (light/dark) conditions. Regenerated plants were transferred to a glass flask with rooting medium N6I (Chu 1978) supplemented with 2 mg l^−1^ indole-3-acetic acid. Seedlings with a well-developed root system were transferred into pots (soil to sand 3:1) and grown to maturity under controlled conditions in the greenhouse on a 16 h/8 h (light/dark) photoperiod. Immature zygotic embryos from each donor plant were plated on all the IM tested (T1–T9); however, regenerants for each trial were not obtained for every donor plant. Between 0 and 12 regenerants were obtained per trial for different donor plants. Finally, regenerants from a single donor plant representing all in vitro culture conditions tested (T1–T9) were selected for analysis. After all, forty-five regenerants originating from one donor plant were prepared for analysis. Five regenerants from each trial (T1–T9) were tested.

**Table 1:** Conditions of triticale plant regeneration on induction media via somatic embryogenesis

Trial	T1	T2	T3	T4	T5	T6	T7	T8	T9
CuSO_4_ × 5H_2_O (μM)	0.1	0.1	0.1	5	5	5	10	10	10
AgNO_3_ (μM)	0	10	60	60	0	10	10	60	0
Incubation time (days)	35	42	49	42	49	35	49	35	42

### DNA extraction and metAFLP procedure

The leaves of young seedlings of 20 donor plants and 45 regenerants were grounded in liquid nitrogen. The extraction of DNA was performed using the DNeasy MiniPrep Kit (Qiagen, Hilding, Germany) according to the procedure recommended by the manufacturer. The amount of DNA was estimated spectrophotometrically (Nanodrop, Thermo Fisher Scientific, Wilmington, USA) at A_260/280_ ∼ 1.8 and A_260/230_∼ 2.0. DNA quality and integrity were tested in a 1.2% agarose gel by staining DNA fragments with ethidium bromide. For the metAFLP technique, two samples of genomic DNA (2 × 500 ng) were prepared from each plant.

Obtaining DNA band profiles by metAFLP was performed for donor plants and regenerants according to the methodology described previously (Bednarek et al. 2007) and then modified (Machczyńska et al. 2014). The prepared DNA samples, two from each plant, were subjected to restriction enzyme digestion; the 6-bp cutting enzymes Acc65I and *KpnI* and the 4-bp cutting enzyme *Mse*I were used (NEB Ipswich, Massachusetts, USA). Adapters were ligated onto the digested DNA fragments, and these fragments were then amplified using a polymerase chain reaction (PCR). After pre-selective PCR amplification, products were diluted with sterile distilled water (1∶19 dilutions), of which 1.5 μL was used as the template for selective PCR amplification with radio-labeled primer (labeling with γ^32^P) and unlabeled *Mse*I + YYY (Table 2). Electrophoresis was carried out on 7% polyacrylamide in 1 × TBE buffer. Gels were exposed to X-ray film, then developed by a conventional procedure.

**Table 2:** Arrangement of primers used for selective DNA amplification of triticale donors and regenerants by the metAFLP technique

metAFLP oligomer	Sequence 5’ → 3’
Labeled γ^32^P selective oligonucleotides	
CG-GAC	CA TGC GTA CAG TAC CGA C
CG-GCA	CA TGC GTA CAG TAC CGC A
CG-GGC	CA TGC GTA CAG TAC CGG C
CG-TCG	CA TGC GTA CAG TAC CTC G
CXG-AGA	CA TGC GTA CAG TAC CAG A
CXG-AGC	CA TGC GTA CAG TAC CAG C
CXG-AGG	CA TGC GTA CAG TAC CAG G
CXG-ATG	CA TGC GTA CAG TAC CAT G
CXG-TGC	CA TGC GTA CAG TAC CTG C
CXG-TTG	CA TGC GTA CAG TAC CTT G
CXX-ATT	CA TGC GTA CAG TAC CAT T
CXX-TAA	CA TGC GTA CAG TAC CTA A
Selective oligonucleotides MseI + YYY	
M-CAC	GAT GAG TCC TGA GTA ACA C
M-CGT	GAT GAG TCC TGA GTA ACG T

### Analysis of data obtained by metAFLP

The metAFLP profiles were scored and generated binary presence-absence data matrices (1 for presence, 0 for absence of a band). Two matrices were prepared: one reflected fragments obtained with *Acc*65I/*Mse*I (A) enzymes and the second with *Kpn*I/*Mse*I (K). The binary data from the two matrices were collated and compared. The Acc65I and MseI enzyme pair for cutting genomic DNA provided information regarding changes in DNA sequence and methylation. On the other hand, cutting genomic DNA with KpnI and MseI enzymes provided information on DNA sequence variations (genetic variation). Comparing the two matrices allows DNA fragments to be extracted and describe changes in DNA methylation (epigenetic variation) and creates a third virtual matrix (M). Based on matrices K and M, we calculated the total number of loci generated in the analysis and expected heterozygosity (He) under the assumption of Hardy–Weinberg equilibrium in GenAlex (Excel add-in software). XLSTAT software was applied to agglomeration (UPGMA and dissimilarity Jaccard coefficients).

The quantitative characterization of TCIV was previously described in detail (Bednarek et al. 2007; Machczyńska et al. 2014), with some modifications accompanied by an Excel file capable of carrying out the relevant calculations (Orłowska and Bednarek 2020). This description included formulas for estimating SV, DMV, DNMV, and TCIV also in specific sequence contexts (CG, CHG—symmetrical, and CHH—asymmetrical) in which in vitro culture-induced changes may occur.

The values obtained during the estimation of in vitro culture-induced changes were analyzed using analysis of variance (one-way ANOVA) and ANOVA with Brown-Forsythe *F* when the data did not meet the assumptions of the Levene test (lack of homogeneity of variances in the study groups). The means for different metAFLP characteristics (TCIV, SV, DMV, DNMV) were compared with each other, also in different sequence contexts (CHH, CG, CHG) for regenerants obtained under different in vitro conditions (trials T1–T9). Multiple comparisons were checked with the Games-Howell post hoc test when the homogeneity of variance was violated. ANOVA was performed using XLSTAT software.

## Results

Twenty donor plants that were the generative progeny of a regenerant obtained through androgenesis showed no morphological differences from each other. Only one donor plant out of twenty from which immature zygotic embryos were obtained for the somatic embryogenesis process yielded 45 regenerants in all in vitro culture variants tested (T1–T9). The regenerants were morphologically identical to the donor plant independently of in vitro plant regeneration conditions (trials T1–T9).

The metAFLP resulted in fully stable and reproducible banding patterns amplified for donors and regenerants. Four hundred forty-four bands amplified with 13 primer pairs based on the KpnI/MseI (K) metAFLP platform were evaluated for donor plants. The Acc65I/MseI – KpnI/MseI (M) data reflecting DNA methylation changes resulted in 34 bands. There were 426 unique bands for donors derived under specific trial conditions and related to sequence variation. In contrast, a unique banding pattern related to DNA methylation change was exhibited by 16 signals (Table 3). The metAFLP analysis (with 11 primer pairs) concerning regenerants revealed the presence of 213 related to sequence variation and 39 to DNA methylation change; 186 and 12 were unique for regenerants representing selected trials. The He was lower for donors (0.007 for K and M) than for the regenerants set (0.014 for K and M markers) (Table 3). The polymorphic loci (%P) percentage was slightly lower for the K matrix than M for donors. Similar differences in the %P level were observed for K and M matrices for regenerants (Table 1). The %P for regenerants also indicated a lower value for sequence data (K) and a higher value for methylation data (M) (Table 3).

**Table 3:** Total band patterns data for donors and regenerants for sequence (K) and methylation (M) pattern change markers

	Donors		Regenerants	
Characteristics	K	M	K	M
No. bands	444	34	213	39
No. private bands	426	16	186	12
Mean heterozygosity (He)	0.007	0.007	0.014	0.014
Standard error of mean He	0.002	0.002	0.004	0.004
Polymorphic loci (%P)	3.04	3.26	5.12	8.27
Standard error of %P	0.011	0.011	0.022	0.022

**Fig. 1:**
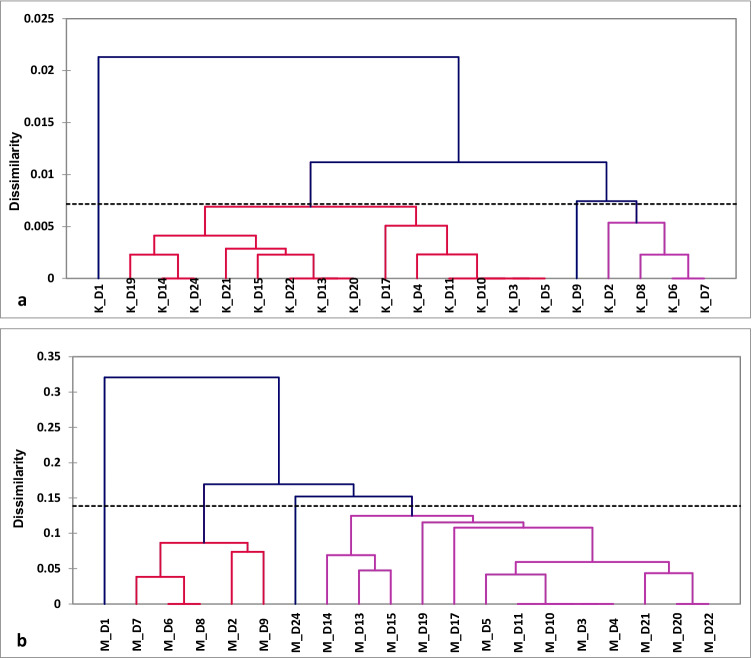
Agglomeration analysis (UPGMA, Jaccard) based on sequence (K) (**a**) and virtual markers related to DNA methylation differences (M) (**b**) evaluated for donors

Agglomeration analysis of donor plants based on (K) and (M) data (Fig. 1a , b) shows donor plants exhibited higher variation based on M than on K. In both analyses, a single donor plant was somewhat apart from the other samples, which were more uniform but still formed three additional clusters.

**Fig. 2:**
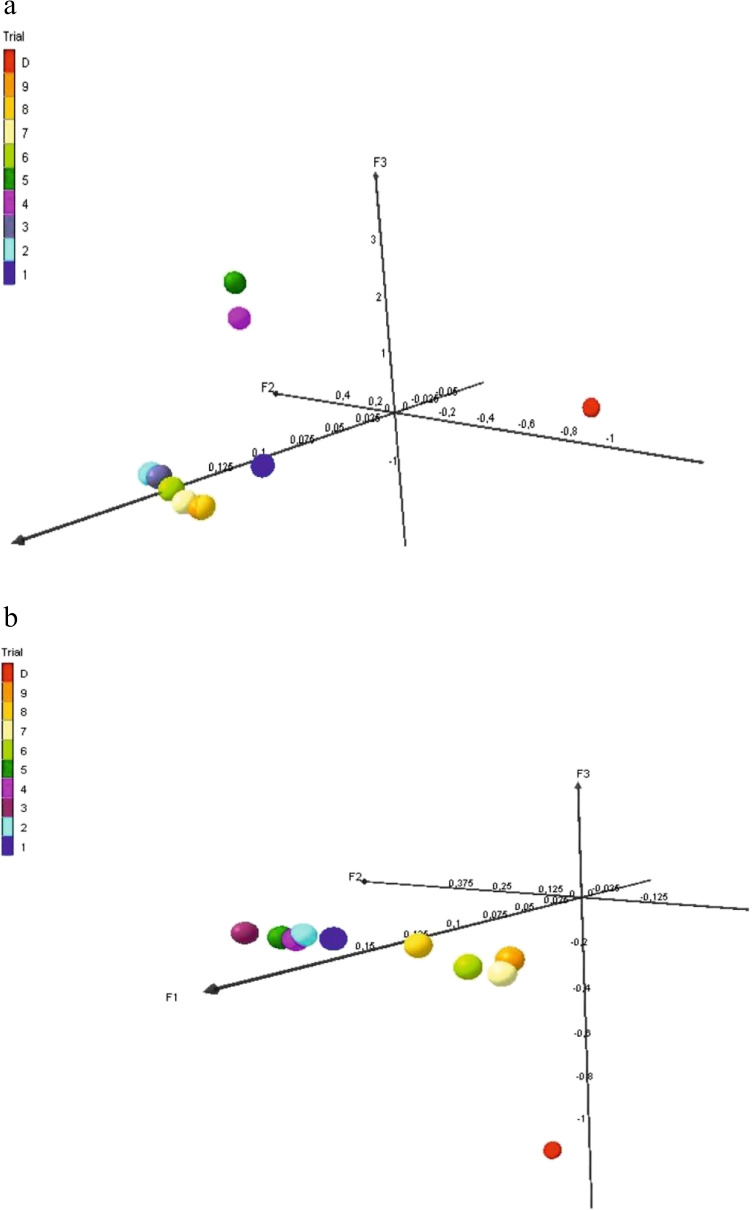
Visualization of factor analysis of molecular data based on sequence (K) (**a**) and virtual markers related to DNA methylation differences (M) (**b**) evaluated for donor D and its regenerants derived via somatic embryogenesis in the T1–T9 trials

Factor analyses carried on K and M markers are congruent with the UPGMA analyses (Fig. 2a, b), where a single donor plant is apart from the other samples, possibly forming clusters encompassing individuals from different trials.

The metAFLP quantitative characteristics (Table 4) showed that the highest level of TCIV was observed for trial T2, with the lowest concentration of copper ions, intermediate levels of silver ions, and the intermediate incubation time of immature zygotic embryos on the IM (Table 1). In contrast, the highest level of copper ions applied, the intermediate level of silver ions, and the highest incubation time resulted in the slightest changes induced by the in vitro culture (T7). Among the components of TCIV, SV was the highest, and maximum and minimum levels of DNA sequence changes were recorded for regenerants from T2 and T7 trials, similarly to TCIV. However, DNA methylation changes were lower than SV and amounted to about 6% for DMV and 1% for DNMV. Furthermore, DMV was favored by the highest levels of copper and silver ions and the intermediate incubation time of the explants on the inducing media (T8), whereas the slightest demethylation events were recorded for the T2–T4 trials at the lowest copper and intermediate and highest silver ion concentrations; the incubation time of immature embryos on the IM for these trials was intermediate and the longest. Similar to DMV, the fewest de novo DNA methylation events were observed for T2–T4 and the highest for T5–T9 (Table 4).

**Table 4:** Mean values for in vitro induced variation and its components for triticale regenerants, including grouping trials by the post hoc test

	metAFLP characteristics^††^			
Trial^†^	TCIV	SV	DMV	DNMV
T1	45.55^a^	38.45^a^	5.97^b^	1.12^a^
T2	45.62^a^	38.57^a^	5.93^b^	1.11^a^
T3	45.47^a^	38.43^a^	5.93^b^	1.11^a^
T4	45.40^a^	38.35^a^	5.93^b^	1.11^a^
T5	45.10^ab^	38.01^ab^	5.97^b^	1.12^a^
T6	45.10^ab^	38.01^ab^	5.97^b^	1.12^a^
T7	44.36^b^	37.29^b^	5.95^b^	1.12^a^
T8	44.84^b^	37.39^b^	6.33^a^	1.12^a^
T9	44.69^b^	37.32^b^	6.26^ab^	1.12^a^
Levene’s test, *p*	0.037	0.027	0.0001	0.0001
Browne-Forsythe *F*-ratio	8.750	15.534	27.199	1.160
*p*	0.0001	0.0001	0.0001	0.429

^†^T1, control; T2–T9, experimental trials

^††^The TCIV, SV, DMV, and DNMV characteristics reflect tissue culture-induced variation, sequence variation, demethylation, de novo methylation, respectively

The a, b and c superscript letters indicate Games-Howell grouping

According to Levene’s test, the assumption of homogeneity of variance for trials in each metAFLP characteristic was violated (Table 4). ANOVA indicated differences between TCIV, SV, and DMV trials, while no differences were observed for DNMV (Table 4). Games-Howell’s post hoc analysis for TCIV trials revealed that trials T1–T4 and T7–T9 compose separate groups; each group contains a trial with the highest or lowest in vitro induced variation level. Similar to TCIV, the Games-Howell test noted inter-trial significant differences for SV. For changes concerning DNA methylation, significant differences were noted only for DMV. The post hoc Games-Howell test indicated a homogeneous group that included regenerants from T1–T7 trials and distinct regenerants representing trial T8. Trial 8 included the regenerants with the highest DMV.

ANOVA and grouping according to the Games-Howell post hoc test indicated significant differences between individual metAFLP characteristics concerning symmetric and asymmetric sequence contexts. For SV and DMV, the most significant changes were observed in the symmetric CHG context and the least in the symmetric CG context. On the other hand, in the case of changes concerning DNMV, no such changes were observed in the symmetric CG context, and the highest DNMV was observed in the asymmetric CHH context (Table 5).

**Table 5:** The results of ANOVA statistics and the Games-Howell post hoc tests demonstrating differences between DNA sequence contexts for the metAFLP characteristics

	metAFLP characteristics		
Methylation context	SV	DMV	DNMV
CHG	21.55^a^	4.44^a^	0.37^b^
CG	6.21^c^	0.45^c^	0.00^c^
CHH	10.21^b^	1.13^b^	0.76^a^
Levene’s test, *p*	0.0001	0.0001	0.0001
Browne-Forsythe *F*-ratio	26,015.670	22,620.932	439,028.173
*p*	0.0001	0.0001	0.0001

The a, b and c superscript letters indicate Games-Howell grouping

**Fig. 3:**
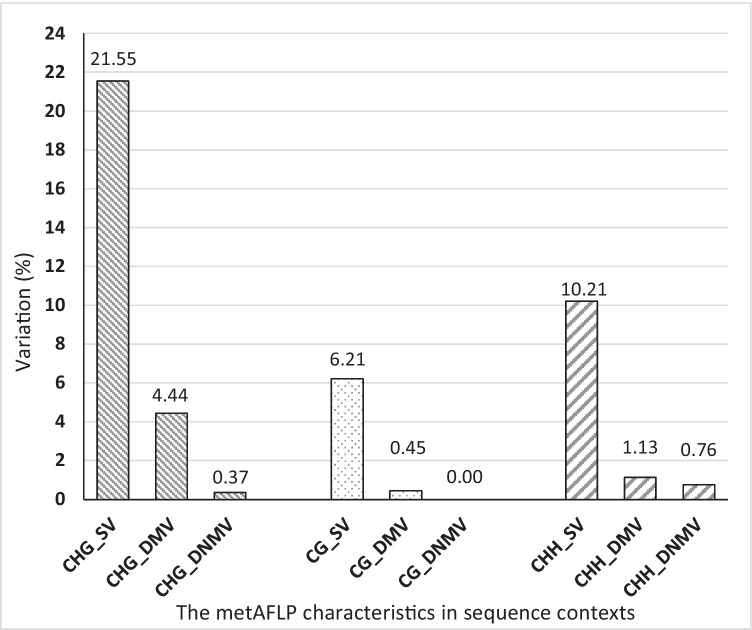
Level of changes identified in regenerants across different metAFLP characteristics and sequence contexts

A detailed examination of the differences between the regenerants belonging to the different trials in the different characteristics of the metAFLP considering the CG, CHG, and CHH contexts showed that a higher variation was observed in the DNA sequence in the CHG context (21.55%) and the least in the CG (6.21%) (Fig. 3). Subsequently, between 4 and 0.45% of the changes were related to DMV in the CHG and CG contexts, and the least number of changes was related to DNMV (0–0.76%).

According to Levene’s test, the assumption of homogeneity of variance for almost all tested metAFLP characteristics and sequence contexts violated the assumptions (*p* < 0.0001), apart from SV_CHG. Thus, ANOVA with a Tukey HSD post hoc test for SV_CHG indicated differences between trials. T1–T3, T4–T6, and T7–T9 trials are divided into separate groups. In the cases of SV_CG and DMV_CG, ANOVA with a Games-Howell post hoc test was used. This analysis for SV_CG indicated differences between regenerants belonging to trials T1-T4, T8, and T9. The same analysis was conducted for DMV_CG grouped regenerants from trials T1–T7, showing their distinctiveness from regenerants belonging to trials T8 and T9 (Table 6). Differences between regenerants obtained in separate in vitro culture conditions for the other metAFLP characteristics and sequence contexts were insignificant.

**Table 6:** ANOVA statistics for metAFLP characteristics considering sequence contexts (CHG, CG) for regenerants obtained in various in vitro conditions (trials T1–T9) with post hoc tests

Trial	metAFLP characteristics in methylation contexts		
	Sequence variation		Demethylation
	SV_CHG	SV_CG	DMV_CG
T1	22.02^a^	6.20^c^	0.37^b^
T2	22.20^a^	6.20^c^	0.36^b^
T3	22.05^a^	6.20^c^	0.36^b^
T4	21.98^ab^	6.20^c^	0.36^b^
T5	21.48^b^	6.21^bc^	0.37^b^
T6	21.48^b^	6.21^bc^	0.37^b^
T7	20.87^c^	6.22^bc^	0.37^b^
T8	20.96^c^	6.23^a^	0.73^a^
T9	20.88^c^	6.23^a^	0.73^a^
Levene’s test, *p*	0.679	0.0001	0.0001
ANOVA *F*-test	23.214		
*p*	0.0001		
Browne-Forsythe *F*-ratio		6.951	2807.620
*p*		0.002	0.0001

The a, b and c superscript letters indicate grouping trials by post-hoc test

## Discussion

Regenerating plants via in vitro cultures is often affected by TCIV (Orłowska et al. 2021a), possibly due to in vitro culture conditions (Bednarek and Orłowska 2019). Such variation was also observed in triticale regenerants obtained by SE, where somaclones/regenerants differ in resistance to the *Fusarium* head blight (Góral and Arseniuk 1997), showing the variation of the mitochondrial atp6 gene region (Schmidt et al. 1996), or showing confirmed rearrangements in the mtDNA (Weigel et al. 1995). The use of the metAFLP method to study variation induced by tissue culture allowed the estimation of TCIV concerning changes in the sequence and methylation of genomic DNA (Machczyńska et al. 2014). Running experiments with the metAFLP technique require the appropriate research material, consisting of selected donor plants and their regenerants (Bednarek et al. 2007). Such material can attribute the observed changes to the action of in vitro culture. Hence, in the present study, evaluating the level and characteristics of variation in triticale regenerants obtained under different in vitro conditions (T1–T9), donor plants were used, which are the generative progeny of the regenerant obtained by androgenesis. All obtained donor plants showed no phenotypic changes and were identical to the initial plant, indicating their genetic uniformity. This result was also confirmed at the level of molecular patterns obtained with the metAFLP technique. We obtained donor plants that revealed as little as 3.04% and 3.26% of polymorphic metAFLP loci concerning sequence and methylation changes, respectively. Also, the expected heterozygosity confirmed a low level of polymorphism of donor plants in the case of both matrices K and M. Cluster analysis showed that donors’ genetic and epigenetic dissimilarities varied depending on whether genetic or methylation data were analyzed, indicating higher variation regarding changes in DNA methylation (0.7% K vs. 14% M). One plant was randomly selected from the pool of donor plants, and regenerants derived from this plant were included in the analysis to avoid the impact of pre-existing variation (Flinn et al. 2020) on TCIV. This procedure avoided disturbances in TCIV that could result from potential variations introduced by the donors.

The evaluation of molecular data for regenerants obtained under different in vitro culture conditions showed that He and %P for regenerants were higher than for donors, which was expected due to tissue culture-induced stress. Furthermore, in regenerants also, more polymorphic fragments were observed for methylation data. Visualization of the factor analysis results for regenerants illustrated the clustering of regenerants forming individual trials and therefore obtained under different in vitro *culture* conditions. For sequence and DNA methylation data, the donor plant was located at the periphery of the regenerants. In contrast, the regenerants formed two groups, each reflecting Cu and Ag ion supplementation and modifications regarding the incubation time of immature zygotic embryos on IM. Thus, pointing to the applied modifications of the substrates and the conditions for obtaining regenerants as those that can influence the resulting changes detected at the molecular level.

The metAFLP analysis performed for triticale regenerants obtained in immature zygotic embryo cultures indicated a high level of TCIV ranging from 44.36 to 45.55%. The result obtained is higher than the TCIV observed in barley regenerants (8.74%) acquired in the same type of tissue culture (Orłowska 2021). However, the results received for TCIV do not generally differ from the level of changes induced in plant tissue culture in triticale regenerants derived by androgenesis (Orłowska et al. 2022), although they are slightly lower (45.13 somatic embryogenesis vs. 51.84 androgenesis). Such significant differences in TCIV levels for regenerants for two different kinds of grain cereal may be due to the genome specificity of each species. Barley is a self-pollinating, diploid species with 2n = 14 chromosomes and a genome size of 5.1 Gbp (Sato 2020). On the other hand, triticale is a synthetic allopolyploid cereal species that combines wheat and rye chromosomes in its genome. When the parental genomes fuse to form a complex allopolyploid genome, rearrangements can occur in the genomes of the parental forms (Ma and Gustafson 2008), which can result in instability of the hybrid genome (Oleszczuk et al. 2019). Also, we can see that manipulation of the concentration of copper and silver ions in the IM is reflected in the level of TCIV, which is highlighted in the ANOVA results.

The presence of copper ions in in vitro culture media, whether in induction or regeneration ones, seems natural, given the importance of this micronutrient in plant function (Yruela 2009). Furthermore, copper is present in more than 100 enzymes involved in crucial life processes of plants (Burkhead et al. 2009); obtaining plants through in vitro cultures may indirectly lead to sequence variation in the genome of regenerants (Orłowska et al. 2021b). The highest level of TCIV observed here was for the T2 trial, where the lowest level of CuSO_4_ × 5H_2_O (0.1 μM) was used. In contrast, the highest concentration of CuSO_4_ × 5H_2_O (10 μM) resulted in the lowest observed TCIV level. These results are consistent with data for triticale regenerants obtained by androgenesis (Orłowska et al. 2022).

The variation induced in plant tissue cultures is genetic and epigenetic. Genetic variation, i.e., changes in DNA sequence, had the highest contribution to TCIV. Similarly, the highest contribution of SV to TCIV was observed in regenerants obtained by androgenesis (Machczyńska et al. 2015; Orłowska et al. 2022) and somatic embryogenesis (Machczyńska et al. 2015), although in the study by Machczyńska and co-authors, the level of SV was lower and oscillated around 19%. The in vitro culture conditions with the lowest CuSO_4_ × 5H_2_O concentration showed the most remarkable genetic changes. It cannot be excluded that the minimum amount of copper does not provide an adequate amount of copper cofactor for the superoxide dismutase Cu/Zn-SOD.

Consequently, the dysfunction of the enzyme and oxidative stress, causing mutations in DNA (Poetsch 2020), may be elevated. Apart from SV, epigenetic changes were also observed in the regenerants, which concerned a decrease in DNA methylation (demethylation) and an increase in genomic DNA methylation compared to the donor plants; these changes were described as de novo methylation. Changes in up- (DNMV-3.34%) or down- (5.15%) methylation were a lot smaller than changes in the sequence variation. Furthermore, differences between regenerants obtained under different in vitro culture conditions were noted only for DMV. ANOVA did not reveal differences between trials concerning DNMV. Regenerants from the T3–T4 trials had the lowest DMV in the presence of a minimal concentration of CuSO_4_ × 5H_2_O.

In contrast, the highest copper ion concentration led to the most significant changes in DMV. This data is only partially consistent with the results for regenerants obtained by anther cultures. While concordance was observed for in vitro culture conditions leading to minimal DMV levels, the highest DMV levels in regenerants obtained by androgenesis were associated with minimal CuSO_4_ × 5H_2_O concentrations. It appears that the high DMV associated with maximum CuSO_4_ × 5H_2_O levels may reflect the action of copper as a factor responsible for ROS production. Elevated levels of ROS may lead to oxidative changes in methylated cytosine (Kurek et al. 2019) and, through repair mechanisms, to its removal (Zhu 2009) and consequently to a decrease in genomic DNA methylation.

Studies on the effect of AgNO_3_ on the regeneration of plants show both positive (Hassan and Islam 2021) and negative (Anantasaran and Kanchanapoom 2008) effects of Ag ions on the production of regenerants. The present work combined the maximum and minimum levels of TCIV with moderate supplementation of IM with AgNO_3_ (10 μM). The same was observed in the case of SV. Regenerants with the lowest level of DMV were derived in trials with moderate and the lowest level of AgNO_3_. Also, the highest level of DMV was observed in regenerants acquired in the presence of the highest value of AgNO_3_ (60 μM). However, one cannot exclude the function of silver as a moderator and mediator in mediation or moderation analyses, where silver concentration as another third variable may influence the regression model.

The variation observed in DNA regenerants assigned to particular sequence contexts was the highest in CHG and CHH contexts. It is not fully congruent with what was observed for triticale regenerants obtained by androgenesis (Orłowska et al. 2022), where the CHG and CG contexts were the most affected. This result is slightly different from the data for plant genomes, where most changes concerning DNA methylation are related to the symmetric context CG for regenerants derived under varying in vitro culture conditions. Here was shown that significant differences between regenerants were recorded for SV in the symmetric CHG (SV_CHG) and CG (SV_CG) contexts and for DMV in the CG context (DMV_CG). Furthermore, for triticale regenerants obtained by androgenesis, differences were recorded for sequence variation (Pachota et al. 2022). Finally, comparing the presented data with the results for barley, we observed that in the genome of barley regenerants received both by somatic embryogenesis and androgenesis, the changes between regenerants concerned all the sequence contexts (Orłowska 2021).

Analyzing different in vitro culture conditions for the changes assigned to SV in the CHG and CG contexts and DMV in the CG context, it can be seen that the highest level of SV in the CHG context is combined with minimal supplementation with copper ions and with the full range of silver concentrations and the full range of days of incubation of immature zygotic embryos on IM. This arrangement of data also applies to SV in the CG context and generally replicates the variation analysis performed for TCIV and SV without splitting the data into methylation contexts. In contrast, DMV in the CG context was highest in maximum CuSO_4_ × 5H_2_O concentration (T8, T9) and the presence (60 μM) (T8) and absence (T9) of Ag ion supplementation. Furthermore, the incubation time of immature zygotic embryos on IM where the highest DMV was detected in the CG context was the lowest (37 days, T8) and moderate (42 days, T9). In contrast, differences between trials for the CG context concerning DMV were not observed for triticale regenerants obtained by androgenesis (Pachota et al. 2022). This relationship between in vitro culture conditions and DMV was similar to an analysis where demethylation changes were studied without considering methylation contexts. Therefore, an analysis that considers the level of variation for regenerants from different trials concerning sequence contexts potentially typifies copper ions as a factor that may influence the observed changes. However, other analyses would be required to determine how copper may affect SV and DMV in symmetric CHG and CG contexts.

## Conclusions

The emergence of TCIV has an impact on the production of triticale regenerants from immature zygotic embryo cultures; furthermore, the magnitude of this variation seems to be influenced by the tissue culture conditions. The modifications that were noticed were impacted by the addition of copper salts to IM. However, the analyses carried out do not suggest the relevance of supplementation with silver salts or the influence of time as factors that can determine genetic and epigenetic alterations in triticale regenerants, at least within the scope of elementary statistical tests. Additionally, an in-depth investigation of the metAFLP technique’s molecular data revealed certain DNA sequence contexts (CHG and CG) in which sequence-related alterations (SV_CG, SV_CHG), as well as DNA demethylation (DMV_CG), were noted. Therefore, we have a tendency to believe that altering copper ion supplementation in the IM affects the balance of metabolic pathways and causes TCIV.

## Data Availability

All data generated during this study are included in this published article.
